# Metabolic and bariatric surgeries in individuals with obesity and BMI of 30–35 kg/m^2^: systematic review

**DOI:** 10.1590/1806-9282.2025D714

**Published:** 2025-06-02

**Authors:** Antonio Silvinato, Idevaldo Floriano, Wanderley Marques Bernardo

**Affiliations:** 1 Brazilian Medical Association, Evidence-Based Medicine – São Paulo (SP), Brazil.; 2 Universidade de São Paulo, Faculty of Medicine – São Paulo (SP), Brazil.

## INTRODUCTION

The association between obesity, type 2 diabetes mellitus (T2DM), and cardiovascular disease (CVD) is well-established^1^. Obesity contributes to the pathogenesis of these conditions through risk factors such as hypertension, dyslipidemia, and glucose intolerance or T2DM, as well as exerting independent effects by promoting systemic inflammation, increasing sympathetic tone, and inducing a prothrombotic state^2^.

With the increasing prevalence of obesity, a proportional rise in the number of individuals with T2DM and CVD is expected^3,4^. According to a study funded by the National Council for Scientific and Technological Development (CNPq), projections indicate that by 2030, 68% of the Brazilian population will be overweight, with obesity affecting 26% of individuals^5^.

Bariatric surgery exerts significant effects on glucose homeostasis by promoting weight loss and triggering a cascade of metabolic and endocrine adaptations^6,7^. Although the underlying mechanisms are not yet fully elucidated, weight reduction is frequently considered the primary driver of improved insulin sensitivity. However, evidence suggests that bariatric surgery also modulates glucose homeostasis through weight-independent mechanisms, including enhanced postprandial secretion of gut hormones such as glucagon-like peptide-1 (GLP-1), peptide YY, and glucose-dependent insulinotropic polypeptide, even before substantial weight loss occurs^7,8-13^. These hormonal changes enhance pancreatic beta-cell function and insulin secretion, supporting the integration of bariatric surgery into the therapeutic paradigm for T2DM^14,15^.

In light of this evidence, the concept of "metabolic surgery" has emerged, referring to the use of bariatric procedures specifically for diabetes treatment, including in individuals with mild obesity or overweight. The primary surgical techniques employed in this context include Roux-en-Y gastric bypass (RYGB), vertical sleeve gastrectomy (VSG), biliopancreatic diversion with duodenal switch (BPD-DS, typically indicated for severe obesity and refractory T2DM), and one-anastomosis gastric bypass (OAGB or mini-bypass).

## OBJECTIVE

The aim of this study was to assess the benefits and harms of metabolic and bariatric surgery (MBS) compared to lifestyle modification and pharmacological treatment in individuals with obesity and a body mass index (BMI) of 30–35 kg/m^2^ through a systematic review and, if sufficient common data are available, a meta-analysis of randomized controlled trials (RCTs).

## METHODOLOGY

This assessment is based on scientific evidence obtained through a systematic review of the literature. When applicable, its conclusions may include a meta-analysis of common outcomes across the included studies. The description of the systematic review methodology follows the standardized checklist items of the Preferred Reporting Items for Systematic Reviews and Meta-Analyses (PRISMA) statement^12^. The protocol was registered in PROSPERO {[PROSPERO (york.ac.uk)]}, with the registration number CRD420251013080.

### Eligibility criteria

The eligibility criteria define the specific elements required to address the clinical question outlined in the objectives of this assessment. They establish the scientific rigor and consistency necessary for study inclusion, as well as the primary reasons for excluding retrieved evidence.

### Study inclusion criteria

- Patients with class 1 obesity (BMI 30–34.9 kg/m^2^);
- Treated with MBS;
- Compared with lifestyle modification and pharmacological management (medical therapy);
- Outcomes: weight loss, BMI reduction, T2DM remission, insulin resistance, and metabolic control;
- RCTs;
- No restrictions on publication period or language;
- Abstracts with data or full-text articles.

### Exclusion criteria

The following studies were excluded:

- Non-randomized clinical trials;
- Systematic reviews with or without meta-analysis;
- Narrative reviews;
- Observational studies and/or case series;
- Studies including adolescents or pregnant women;
- Studies reporting only surrogate endpoints;
- Studies lacking extractable data on relevant outcomes (absolute numbers and/or means).

### Evidence search

The searches were conducted in the following databases of published scientific information: Medline/PubMed, Cochrane Central Register of Controlled Trials (CENTRAL), LILACS, and ClinicalTrials.gov (CT.gov) for unpublished registered studies.

Additional manual searches were performed in the reference lists of included studies and other relevant sources. The database search was conducted up to February 2025.

The search strategies used for each database were as follows:

- MEDLINE/PubMed—(Bariatrics OR Bariatric OR Gastroplasty OR Gastroplasties OR Bypass) AND (Obesity OR Overweight) AND (clinicaltrial[Filter] OR randomizedcontrolledtrial[Filter])
- Cochrane CENTRAL—(Bariatrics OR Bariatric OR Gastroplasty OR Gastroplasties OR Bypass) AND (Obesity OR Overweight)
- LILACS—(Bariatrics OR Bariatric OR Gastroplasty OR Gastroplasties OR Bypass) AND (Obesity OR Overweight)
- ClinicalTrials.gov (CT.gov)—(Bariatrics OR Bariatric OR Gastroplasty OR Gastroplasties OR Bypass) AND (Obesity OR Overweight)

### Study selection and data extraction process

The evidence retrieved from the consulted databases was initially selected based on the title and abstract, aiming to meet the eligibility criteria. Studies identified in this initial screening then had their full texts accessed to confirm their eligibility.

The retrieval and evaluation of titles and abstracts were conducted independently and in a blinded manner by two researchers with expertise in systematic reviews (A.S. and I.F.), following the inclusion and exclusion criteria. Subsequently, the selected articles underwent a critical appraisal to determine their inclusion in the review. In cases of disagreement between investigators regarding study selection, a third reviewer (W.M.B.) was consulted.

The following data were extracted from eligible studies: first author's name and year of publication, study population, intervention and comparator methods, and follow-up duration. Relevant outcome data were extracted as absolute event numbers or means and/or medians, along with their respective standard deviations or 95% confidence intervals, depending on the type of outcome.

### Risk of bias and quality of evidence

Two independent reviewers assessed the risk of bias in the included studies using the Cochrane Risk of Bias Tool for randomized trials (RoB 2)^16^, along with additional key elements, classifying the risk as high, moderate, or low. Each domain was categorized as free of bias, unclear, or at risk of bias.

Publication bias was evaluated through visual inspection of the funnel plot and Egger's test^17^.

The Grading of Recommendation, Assessment, Development, and Evaluation (GRADE)^18^ criteria were applied to assess the certainty of the estimated effect in pooled evidence, classifying the quality of evidence into four levels: high, moderate, low, and very low. Two reviewers evaluated the risk of bias, inconsistency, indirectness, imprecision, and publication bias for all reported outcomes. The quality of evidence was assessed using the "Guideline Development Tool" (GRADEpro GDT)^19^ and presented in GRADE evidence profiles and summary of findings tables, employing standardized terminology.

### Method of analysis and synthesis of results

Data were analyzed following the intention-to-treat principle, and the most recent available follow-up data were included for each trial.

Categorical outcome results were expressed as risk difference (RD) between the intervention and control groups using the Mantel–Haenszel method. If the RD between groups was statistically significant (95% confidence level), it was reported along with the 95% confidence interval (95%CI) and the number needed to treat (NNT) or harm (NNH). For continuous outcomes, results were expressed as mean differences (MD) or standardized mean differences (SMD) if different scales were reported, with 95% confidence intervals.

If multiple studies reported common outcomes, they were pooled through meta-analysis using the "metabin" and "metacont" functions from the "meta" package in R software, version 4.4.2 (R Core Team, 2025, Vienna, Austria)^20^.

### Evidence synthesis and conclusion

The evidence synthesis will present the results directly from the analyses, considering benefits, harms, and the absence of differences between metabolic and bariatric surgeries compared to lifestyle modification and pharmacological treatment (medical therapy).

The conclusions will primarily take into account evidence of at least moderate quality, the presence of an effect—whether beneficial or harmful—and a favorable balance between benefits and harms in patients with obesity and a BMI of 30–35 kg/m^2^.

## RESULTS

The evidence search identified 9,392 studies across the Medline (PubMed), CENTRAL, LILACS, and CT.gov databases. Manual searches and/or gray literature did not reveal any additional studies.

After duplicate removal and screening by title and/or abstract, 45 studies met the pre-established eligibility criteria. The full texts of these 45 studies were accessed for analysis.

Following full-text review, eight RCTs with parallel comparison to lifestyle modification and pharmacological treatment (medical therapy) were included to support the conclusions of this assessment^21-27,29^. Two of these studies^22,24^ originated from the same clinical trials but reported different follow-up periods.

The reasons for excluding the other 37 studies are detailed in Supplementary Figure 1, which presents a flow diagram illustrating the entire process from initial retrieval to final evidence selection.

The key baseline characteristics and details of each included trial are described in Supplementary Table 1.

Regarding the risk of bias in the five pivotal studies included^21-24^: none employed blinded outcome assessment; one study^22^ had a loss to follow-up exceeding 20%, differences in prognostic characteristics between groups that could influence results, and did not conduct an intention-to-treat analysis; another study^23^ did not report a sample size calculation (Table 1).

**Table 1:** Risk of bias in studies.

Risk of bias in randomized clinical trials											
Study	Rand.	Alloc.	D-blind	Eval. blind		Losses	Prog. char.	Outcome	ITT	Sample calc.	Early inter.
Cohen et al.^21^											
Parikh et al^22^											
Chong et al.^23^											
O’Brien et al.^24^											
Liang et al.^25^											
Legend	Low risk of bias				Presence of bias				Uncertain risk of bias		

1 Rand.: randomization; Alloc.: allocation; D-Blind: double-blind; Eval. Blind: blinded evaluator; Losses: losses to follow-up; Prog. Char.: prognostic characteristics; Outcome: outcome assessment; ITT: intention-to-treat analysis; Sample Calc.: sample size calculation; Early Inter.: early intervention.

A summary of the findings and the certainty of the evidence for the main outcomes is presented in Supplementary Table 2.

Cohen et al. in an RCT with a 2-year follow-up, including a total of 100 patients with T2DM, obesity (BMI of 30–35 [calculated as weight in kilograms divided by height in meters squared]), and chronic kidney disease (CKD) stages G1 to G3 and A2 to A3 (early stage—urinary albumin-to-creatinine ratio [uACR] >30 mg/g and estimated glomerular filtration rate >30 mL/min). These patients were randomized 1:1 to receive either "best medical treatment" (n=49) or RYGB (n=51). At the 2-year follow-up, compared to medical treatment, RYGB increased the likelihood of albuminuria remission (albumin-to-creatinine ratio <30 mg/g creatinine) by 21.6% (95%CI 2.8–40.4), with a NNT of 5 (95%CI 2–35), increased the likelihood of CKD remission by 33.4% (95%CI 15.9–50.9), with an NNT of 3 (95%CI 2–6), and reduced BMI (MD −6.96, 95%CI −8.02 to −5.89, p<0.001). There was no difference in metabolic control (HbA1c level below 7%, LDL-C level above 100 mg/dL, systolic BP below 130 mmHg, and diastolic BP below 80 mmHg) or in the risk of serious adverse events (Supplementary Table 3). Moderate quality of evidence.

Parikh et al. conducted an RCT including patients with T2DM, BMI 30–35, and eligibility for bariatric surgery based on the National Institutes of Health (NIH) criteria. Notably, 57 patients were randomized to either intensive medical weight management (MWM) or laparoscopic surgery (bypass, sleeve, or gastric banding, based on patient preference). The primary outcomes assessed at 6 months were changes in insulin resistance (Homeostatic Model Assessment of Insulin Resistance [HOMA-IR]) and diabetes remission (no longer meeting the American Diabetes Association [ADA] criteria for T2DM without the use of diabetes medications). Secondary outcomes included changes in HbA1c, percentage of excess weight loss (%EWL), and BMI. Compared to MWM, at 6 months, surgical treatment increased the likelihood of diabetes remission by 65% (RD=65%, 95%CI 44–86; NNT=2, 95%CI 1–2), reduced HOMA-IR, HbA1c, and BMI (p<0.01 for all comparisons), and increased %EWL (p<0.001). There was no difference in the risk of serious adverse events (Supplementary Table 4). Moderate quality of evidence.

Horwitz et al. conducted a retrospective review of the original cohort from the study by Parikh et al., including patients who transitioned from MWM to surgery. In the original study, 29 patients were randomized to surgery and 28 to MWM. At the 6-month analysis, there was a 23% loss of patients. In the 3-year analysis (Horwitz et al.), with 10 patients migrating from the MWM group to surgery after 6 months, an additional 13% loss occurred, resulting in a final analysis of 30 patients in the surgery group and 14 in the MWM group (who underwent MWM for 6 months followed by usual care).

Therefore, we will analyze only the study by Horwitz et al., which examined the pivotal study and a 5-year follow-up, with a relatively lower loss than the 2016 study. Long-term follow-up data (5 years) were available for 43 of the 57 original patients (75%), including 14 of 18 (77%) non-surgical patients and 29 of 39 (74%) surgical patients. There was a 10-patient migration from the MWM group to surgery after 6 months, with a total loss of 25%. The 29 surgical patients consisted of 18 LSG, 8 RYGB, and 3 laparoscopic adjustable gastric banding (LAGB). The mean follow-up period was 79 months in the surgical group and 88 months in the non-surgical group.

Compared to clinical treatment, surgery increased the likelihood of T2DM remission by 38% (95%CI 20–55; NNT=3, 95%CI 2–5), reduced HbA1c (MD=-1.33, 95%CI −2.34 to −0.31; p=0.01) and BMI (MD=-2.8, 95%CI −4.94 to −0.65; p=0.01), and increased the percentage of weight loss (MD=11.1% [95%CI 5.17–17.02], p<0.001) (Supplementary Table 4). Moderate quality of evidence.

Chong et al. conducted a subgroup analysis of the DSS (Diabetes Surgery Study^28^). This RCT was conducted at four university hospitals in the United States and four in Taiwan, involving 71 participants with mild obesity (BMI 30–35 kg/m^2^). In total, 36 participants were randomly assigned to the RYGB group and 35 to the intensive medical management (IMM) group, with a 2-year follow-up.

IMM consisted of two components: lifestyle modification designed to achieve the maximum possible weight loss and medications to control blood glucose and cardiovascular disease risk factors while facilitating weight loss. At baseline, Taiwanese participants had a lower BMI, were younger, and had a shorter T2DM duration compared to American participants.

The primary outcome assessed was partial or complete T2DM remission, defined as blood HbA1c <6.5% (48 mmol/mol) and <6% (42 mmol/mol), respectively, without any antihyperglycemic medication for at least 1 year. No IMM participant, from either population, achieved partial or complete T2DM remission. In the RYGB group, a substantial proportion of individuals achieved partial or complete remission (57% in Taiwanese participants and 27% in American participants, p=0.08). Overall, at up to 2 years, RYGB compared to IMM increased the likelihood of partial or complete T2DM remission by 39% (RD=39%, 95%CI 23–55; NNT=3, 95%CI 2–4) (Supplementary Table 5). Moderate quality of evidence.

O’Brien et al. conducted an RCT with 80 adults from the general community with mild to moderate obesity (BMI 30–35). Participants were assigned to either a very low-calorie diet program, pharmacotherapy, and lifestyle modification for 24 months (non-surgical group) or LAGB.

Inclusion criteria were age between 20 and 50 years, a BMI between 30 kg/m^2^ and 35 kg/m^2^, and the presence of obesity-related comorbidities (such as hypertension, dyslipidemia, diabetes, obstructive sleep apnea, or gastroesophageal reflux disease), severe physical limitations, or clinically significant psychosocial issues associated with obesity.

After 2 years of follow-up, the surgical group achieved significantly greater weight loss: an average of 21.6% of initial body weight (95%CI 19.3–23.9) and 87.2% of excess weight (95%CI 77.7–96.6). In the non-surgical group, weight loss was 5.5% of initial body weight (95%CI 3.2–7.9) and 21.8% of excess weight (95%CI 11.9–31.6) (p<0.001) (Supplementary Table 6). Moderate quality of evidence.

Metabolic syndrome was initially present in 15 patients (38%) in each group. By the end of the study, this condition was observed in eight patients (24%) in the non-surgical group and only one patient (3%) in the surgical group (p<0.002).

Quality of life showed a significantly greater improvement in the surgical group, with gains in all eight subscores of the Short Form-36, whereas the non-surgical group showed improvement in only three out of eight subscores. The study lacked statistical power to compare the occurrence of adverse events. Moderate quality of evidence.

In the non-surgical group, 33 patients (83%) completed the 2-year follow-up. Of these, 17 (43%) later crossed over to undergo the LAGB procedure at various time points after the 2-year follow-up, ranging from 1 week to 74 months (mean: 24 months), forming the cross-over group.

Outcome data were available for 37 (92.5%) of the surgical patients and 27 (62.5%) of the non-surgical patients after 10 years of follow-up (O’Brien et al.). Among participants who completed the 10-year follow-up, there were no baseline differences between the three subgroups: surgical (n=31), non-surgical (n=10), and cross-over (n=10).

The assessment of adverse events was limited to complications associated with the LAGB procedure, including patients from both the surgical and cross-over groups. Sustained weight loss was more evident in the surgical group, with a mean weight loss of 14.1 kg (SD=7.7) after 10 years, corresponding to 63.4% of excess weight loss (EWL). This result was significantly superior to the non-surgical group, which had a mean weight loss of only 0.4 kg (SD=10.5) (p<0.001).

Among the 57 patients who underwent the LAGB procedure, 17 (30%) experienced proximal gastric dilation, and band removal was necessary in seven (12%) cases. Over the 10-year period, 31 adverse events were recorded among these 57 patients, corresponding to an annual event rate of 5.4%. Low quality of evidence.

Liang et al. conducted an RCT to evaluate the effect of laparoscopic RYGB surgery compared to usual care, with or without exenatide therapy, in obese patients (BMI 28–35) with T2DM and hypertension over a 12-month follow-up. A total of 188 patients were included and randomly assigned into three groups: usual care (Group A), usual care plus exenatide (Group B), and RYGB surgery (Group C).

After 12 months of follow-up, RYGB surgery, compared to usual care, increased the likelihood of T2DM remission by 90% (RD=90%, 95%CI 80–100; NNT=1, 95%CI 1–1) and significantly reduced HOMA-IR, glycated hemoglobin (HbA1c), and BMI (p<0.001 for all comparisons). There was no difference in the occurrence of serious adverse events among the groups at 12 months (NNT=not significant; Supplementary Table 7). Moderate quality of evidence.

## EVIDENCE SUMMARY

In patients with obesity and a BMI of 30 to 35 kg/m^2^, metabolic and bariatric surgeries, compared to lifestyle modification and medical therapy, show the following results:

- Over 5 years, surgical treatment increases T2DM remission by 38% (NNT=3; 95%CI 2–5); reduces HbA1c (MD −1.33; p=0.01); decreases BMI (MD −2.8; p=0.01); and increases the average percentage of weight loss (MD 11%; p<0.001). Moderate quality of evidence.
- At 2 years, Roux-en-Y gastric bypass (RYGB) increases complete or partial T2DM remission by 39% (NNT=3; 95%CI 2–4). Moderate quality of evidence.
- After 2 years, RYGB increases albuminuria remission by 21.6% (NNT=5; 95%CI 2–35) and the probability of early stage CKD remission by 33.4% (NNT=3; 95%CI 2–6). Moderate quality of evidence.
- At 1 year, RYGB increases T2DM remission by 90% (NNT=1; 95%CI 1–1) and significantly reduces HOMA-IR, HbA1c, and BMI. Moderate quality of evidence.
- At 6 months, surgical treatment increases diabetes remission by 65% (NNT=2; 95%CI 1–2); reduces HOMA-IR, HbA1c, and BMI; and increases the %EWL. Moderate quality of evidence.
- At 2 years, adjustable gastric banding (LAGB) results in significantly greater weight loss—on average, 21.6% of initial body weight and 87.2% of excess weight—compared to non-surgical treatment, which leads to a weight loss of 5.5% of initial body weight and 21.8% of excess weight (p<0.001). Moderate quality of evidence.
- Over a 10-year period, LAGB presents an annual adverse event rate of 5.4%. Low quality of evidence.
- No evaluated study demonstrated an increased risk of severe adverse events with metabolic and bariatric surgeries compared to lifestyle modification and medical therapy. Moderate quality of evidence.

## DISCUSSION

Class I obesity (BMI 30–34.9 kg/m^2^) is a well-established condition that can cause or exacerbate multiple medical and psychological comorbidities, reduce longevity, and impair quality of life. Randomized clinical trials specifically designed to study individuals with obesity and a BMI of 30–35 kg/m^2^ who do not achieve substantial or sustained weight loss or improvement in comorbidities through non-surgical methods have demonstrated significant benefits of MBS compared to other approaches.

Our systematic review assessed primary outcomes such as changes in insulin resistance (HOMA-IR), remission of T2DM—defined as the absence of established diagnostic criteria by the ADA without the use of diabetes medications—and the occurrence of complications, providing a comprehensive overview of the efficacy and safety of this intervention. Changes in glycated hemoglobin (HbA1c), %EWL, and BMI variation were considered secondary outcomes.

Our results indicate that, over 5 years, surgery increased T2DM remission by 38% (NNT=3; 95%CI 2–5), reduced HbA1c (MD=-1.33; p=0.01) and BMI (MD=-2.8; p=0.01), and led to a 11% greater weight loss (p<0.001). After 2 years, RYGB increased the rate of complete or partial diabetes remission by 39% (NNT=3; 95%CI 2–4), while adjustable gastric banding (LAGB) resulted in significantly greater weight loss compared to non-surgical treatment (p<0.001). At 1 year, RYGB increased diabetes remission by 90% (NNT=1; 95%CI 1–1) and significantly reduced insulin resistance (Homeostatic Model Assessment of Insulin Resistance—-HOMA-IR), HbA1c, and BMI. No study reported an increased risk of serious adverse events associated with MBS.

In summary, our analysis suggests that MBS is more effective than lifestyle modifications and medical therapy in promoting weight loss and improving glycemic control in patients with obesity and a BMI of 30–34.9 kg/m^2^. RYGB shows the highest rates of diabetes remission in follow-ups of up to 5 years. However, it is essential to recognize the limitations of the available evidence, particularly the moderate-to-low certainty of findings due to the risk of bias in the included studies. Another key limitation is the scarcity of long-term studies (>5 years of follow-up) in this specific patient group. Additionally, the effects of MBS on critical outcomes such as mortality and cardiovascular morbidity remain underexplored, and no studies have assessed its impact on diabetes-related complications.

## CONCLUSION

Considering the current data available in the literature, our findings suggest that MBS is more effective than lifestyle modifications and medical therapy in promoting weight loss and improving glycemic control in patients with obesity and a BMI of 30–34.9 kg/m^2^. RYGB shows the highest rates of diabetes remission in follow-ups of up to 5 years. No increase in the risk of serious adverse events was identified. These findings support the consideration of metabolic and bariatric surgery as a therapeutic option in selected patients.
