# Supplementary material for: Metabolic and bariatric surgeries in individuals with obesity and BMI of 30–35 kg/m2: systematic review
Source: Rev Assoc Med Bras (1992). 2025 Jun 2;71(4):e2025D714. doi: 10.1590/1806-9282.2025D714 (PMC12131899; doi:10.1590/1806-9282.2025D714)
Supplement: Supplementary file 1 [file 1806-9282-ramb-71-04-e2025D714-Suppl01.docx]

**Supplementary Material**

**The selection of studies retrieved from the virtual scientific information databases is detailed in the flowchart below:**

Studies identified in the search

(n = 9,392)

## **Identification**

**Studies screened by title and/or abstract**

**(n = 9,392)**

**Studies selected**

**(n = 45)**

## **Screening**

Total studies excluded = 37

- BMI ≠ 30–35 kg/m² (n = 36)
- Lack of reported outcomes (n = 1, NCT02036138)

**Full-text articles assessed for eligibility (n = 8)**

## **Eligibility**

**Studies included in the qualitative analysis**

**(n = 8)**

## **Included**

**Figure 1.** Flowchart of evidence retrieval and selection.^30^

**Table 1.** Description and data of included studies.

| **Author** | **Year** | **Population** | **Intervention** | **n** | **Comparison** | **n** | **Outcome** | **Follow-up** |
| --- | --- | --- | --- | --- | --- | --- | --- | --- |
| Cohen | 2020^21^ | T2DM, BMI 30–35 | Roux-en-Y bypass | 51 | “Best Medical Treatment”: medications | 49 | BMI, abdominal circumference, lipid profile, blood glucose, renal function | 2 years |
| Horwitz | 2020^27^ | T2DM, BMI 30–35 | Bypass, band, or sleeve | 29 | Medical Weight Management: lifestyle changes, education, diet, physical activity | 28 | Weight loss, BMI, HbA1c, blood pressure, lipid profile | 5 years |
|  | 2016^26^ |  |  |  |  |  | Weight loss, BMI, HbA1c | 3 years |
| Parikh | 2014^22^ |  |  |  |  |  | Weight loss, BMI, abdominal circumference, blood pressure, HbA1c, glucose, insulin, lipid profile | 6 months |
| Chong | 2017^23^ | T2DM, BMI 30–35 | Roux-en-Y bypass | 36 | “Intensive medical management”: lifestyle changes, endocrinologist consultations, medications | 35 | BMI, abdominal circumference, lipid profile, HbA1c | 2 years |
| O'Brien | 2013^29^ | BMI 30–35 | Adjustable gastric banding | 40 | Lifestyle changes, diet, pharmacotherapy | 40 | Weight loss, BMI, blood pressure, HbA1c, glucose, insulin, lipid profile | 10 years |
|  | 2006^24^ |  |  |  |  |  |  | 2 years |
| Liang | 2013^25^ | T2DM, BMI 28–35 | Group C: Roux-en-Y bypass | 31 | Group A: “usual care” (multidisciplinary team, medications)  Group B: “usual care”+exenatide | A: 36  B: 34 | BMI, lipid profile, HbA1c | 1 year |

T2DM: Type 2 diabetes mellitus; BMI: body mass index; HbA1c: glycated hemoglobin; n: sample size.

**Table 2.** Summary of findings—Grading of Recommendations, Assessment, Development, and Evaluation.

Metabolic and Bariatric Surgeries Compared to Medical Treatment for Obesity and BMI 30–35 kg/m²

**Patient and Population:** Obese individuals with BMI 30–35 kg/m²

**Intervention:** Metabolic and bariatric surgeries

**Control:** Medical treatment

| **Outcome**  **№ of participants (studies)** | **Anticipated absolute effects (95%CI)** | | | **Certainty** |
| --- | --- | --- | --- | --- |
|  | **Clinical treatment** | **Metabolic and bariatric surgeries** | **Difference** |  |
| Diabetes remission at 5 years  № of participants: 43  (1 RCT) | 0.0% | 37.9% | RD=38% (95%CI 20–55%);  NNT=3 (95%CI 2–5) | ⨁⨁⨁◯  Moderate^a,b,c,d^ |
| Partial or complete diabetes remission at 2 years  № of participants: 71  (1 RCT) | 0.0% | 38.9% | RD=39% (95%CI 23–55%);  NNT=3 (95%CI 2–4) | ⨁⨁⨁◯  Moderate^a,e^ |
| Diabetes remission at 1 year  № of participants: 67  (1 RCT) | 0.0% | 90.3% | RD=90% (95%CI 80–100%);  NNT=1 (95%CI 1–1) | ⨁⨁⨁◯  Moderate^a^ |
| CKD remission at 2 years  № of participants: 100  (1 RCT) | 49% | 82.4% | RD=33.4% (95%CI 15,9–50.9%);  NNT=3 (95%CI 2–6) | ⨁⨁⨁◯  Moderate^a^ |

CI: confidence interval; RD: risk difference; NNT: number needed to treat; RCT: randomized controlled trial; CKD: chronic Kidney Disease. ^a^There was no assessor blinding. ^b^Losses >20%. ^c^Prognostic characteristics may have influenced the results. ^d^No ITT (intention-to-treat). ^e^No sample size calculation.

**Table 3.** Results of Cohen et al. study^21^.

| **Study** | **Follow-up** | **Albuminuria remission** | | **CKD remission** | | **Metabolic control** | | **BMI** | | **Serious adverse events** | |
| --- | --- | --- | --- | --- | --- | --- | --- | --- | --- | --- | --- |
|  |  | **RYGB** | **Medical Treatment** | **RYGB** | **Medical Treatment** | **RYGB** | **Medical Treatment** | **RYGB** | **Medical Treatment** | **RYGB** | **Medical Treatment** |
| Cohen et al.^21^ | 2 anos | 36/51 | 24/49 | 42/51 | 24/49 | 13/51 | 10/49 | 24.26 (23.51 a 25.01) n=51 | 31.22 (30.47 a 31.98) n=9 | 6/46 | 6/46 |
| RD=21.6%, 95%CI 2.8–40.4%; NNT=5, 95%CI 2–35 | | RD=33.4%, 95%CI 15.9–50.9%; NNT=3, 95%CI 2–6 | | NNT=NS | | MD=−6.96 (−8.02 a −5.89), p<0.001 | | NNT=NS | |  |  |

RD: risk difference; MD: mean difference; NNT: number needed to treat; NS: not significant; BMI: body mass index; RYGB: Roux-en-Y gastric bypass; CKD Remission: composite criterion from the American Diabetes Association, defined as urinary albumin-to-creatinine ratio <30 mg/g creatinine and eGFR >60 mL/min/1.73 m²; Albuminuria Remission: albumin-to-creatinine ratio <30 mg/g creatinine; Metabolic Control: defined as HbA1c level <7%, LDL-C level >100 mg/dL, systolic BP <130 mmHg, and diastolic BP <80 mmHg.

**Table 4.** Results of the studies by Parikh et al.^22^ and Horwitz et al.^27^.

| **Study** | **Follow-up** | **HOMA-IR** | | **Diabetes remission** | | **HbA1c** | | **BMI** | | **%EWL** | | **Serious adverse events** | |
| --- | --- | --- | --- | --- | --- | --- | --- | --- | --- | --- | --- | --- | --- |
|  |  | **Surg. Tx.**  **n=20** | **Clin. Tx.**  **n=24** | **Surg. Tx.** | **Clin. Tx.** | **Surg. Tx.** | **Clin. Tx.** | **Surg. Tx.** | **Clin. Tx.** | **Surg. Tx.** | **Clin. Tx.** | **Surg. Tx.** | **Clin. Tx.** |
| Parikh et al.^22^ | 6 months | 1.8 (1.2) | 4.7 (4.9) | 13 /20 | 0/24 | 6.2 (0.9) | 7.8 (1.7) | 25.9 (2.5) | 31.4 (2.6) | 60.0% (21.1) | 7.4% (12.6) | 0/24 | 0/20 |
|  | | MD: -2.9 (-5.16 to -0.63), p=0.01 | | RD=65% 95%CI 44 a 86%; NNT=2, IC95% 1 a 2 | | MD: -1.6 (-2.45 to -0.74), p<0.001 | | MD: -5.5 (-7.06 to -3.93), p<0.001 | | MD: 52.6 (42.22 to 62.9), p<0.001 | | NNT=NS | |
| Horwitz et al.^27^ | 5 years (crossed over) |  | | n=29 | n=14 | n=27 | n=14 | n=29 | n=14 | n=29 | n=14 |  | |
|  |  |  |  | 11/29 | 0/14 | 6.93 (1.37) | 8.26 (1.80) | 25.8 (3.1) | 28.6 (3.6) | 21.4% (9.4) | 10.3% (8.1) |  |  |
|  | |  | | RD=38%, 95%CI 20–55%; NNT=3, 95%CI 2–5 | | MD: -1.33 (-2.34 to -0.31), p=0.01 | | MD: -2.8 (-4.94 to -0.65), p=0.01 | | MD: 11.1% (5.17 to 17.02), p<0.001 | |  |  |

RD: risk difference; MD: mean difference; NNT: number needed to treat; NS: not significant; Surg. Tx.: Surgical treatment (Laparoscopic Roux-en-Y gastric bypass [RYGB], adjustable gastric banding, or laparoscopic sleeve gastrectomy [LSG]); Clin. Tx.: Clinical treatment (physician-supervised weight management); T2DM was defined according to the American Diabetes Association (ADA) criteria: (1) fasting glucose ≥126 mg/dL, or (2) glucose ≥200 mg/dL at 120 min after a 75 g oral glucose load, or (3) HbA1c ≥6.5%; Diabetes remission: no longer meeting ADA criteria for T2DM without diabetes medications; %EWL: percentage of excess weight loss; HOMA-IR: Homeostatic Model Assessment of Insulin Resistance; HbA1c: glycated hemoglobin; BMI: body mass index.

**Table 5.** Results of Chong et al. study.^23^

| **Study** | **Follow-up** | **Partial or complete remission of T2DM** | |
| --- | --- | --- | --- |
|  |  | **RYGB** | **IMM** |
| Chong et al.^23^ | 2 years | 14/36 | 0 /35 |
| RD=39%, 95%I 23 a 55%; NNT=3, 95%CI 2 a 4 | |  |  |

IMM: Intensive medical management; T2DM: type 2 diabetes mellitus; RYGB: Roux-en-Y gastric bypass.

**Table 6.** Results of O'Brien et al. study^24^.

| **Study** | **Follow-up** | **Weight, Kg** | | **BMI** | | **%EWL** | | **Serious adverse events** | |
| --- | --- | --- | --- | --- | --- | --- | --- | --- | --- |
|  |  | **Band** | **Clin. Tx** | **Band** | **Clin. Tx** | **Band** | **Clin. Tx** | **Band** | **Clin. Tx** |
| O'Brien et al.^24^ | 2 years | 74.5 (72.4–76.7) | 89.5 (80.5–98.5) | 26.4 (25.6–27.2) | 31.5 (30.6–32.4) | 87.2 (77.7–96.6) | 21.8 (11.9–31.6) | 0/40 | 0/40 |
| p<0.001 | | p<0.001 | | p<0.001 | | NNT=NS | |  |  |

Clin. Tx.: clinical treatment; NNT: number needed to treat; NS: not significant; BMI: body mass index; %EWL: percentage of excess weight loss.

**Table 7.** Results of Liang et al. study.^25^

| **Study** | **Follow-up** | **HOMA-IR** | | **Diabetes remission** | | **HbA1c** | | **BMI** | | **Serious adverse events** | |
| --- | --- | --- | --- | --- | --- | --- | --- | --- | --- | --- | --- |
|  |  | **RYGB**  **n=36** | **Usual Care**  **n=31** | **RYGB** | **Usual Care** | **RYGB** | **Usual Care** | **RYGB** | **Usual Care** | **RYGB** | **Usual care** |
| Liang et al.^25^ | 1 year | 1.50±0.09 | 2.33±0.24 | 28/31 | 0/36 | 5.98±0.30 | 8.14±0.27 | 24.51±0.91 | 30.38±1.66 | 0/31 | 0/36 |
| MD: -0.83 (-0.91 to -0.74), p<0.001 | | RD=90%, 95%CI 80 to 100%; NNT=1, 95%CI 1 to 1 | | MD:  -2.16 (-2.30 to -2.01), p<0.001 | | MD:  -5.87 (-6.51 to -5.22), p<0.001 | | NNT=NS | |  |  |

RD: risk difference; MD: mean difference; NNT: number needed to treat; NS: not significant; HOMA-IR: Homeostatic Model Assessment of Insulin Resistance; HbA1c: glycated hemoglobin; BMI: body mass index; RYGB: Roux-en-Y gastric bypass.

**GRADE Working Group grades of evidence**

**High certainty:** we are very confident that the true effect lies close to that of the estimated effect.

**Moderate certainty:** we are moderately confident in the effect estimate: the true effect is likely to be close to the estimate of the effect, but there is a possibility that it is substantially different.

**Low certainty:** our confidence in the effect estimate is limited: the true effect may be substantially different from the estimate of the effect.

**Very low certainty:** we have very little confidence in the effect estimate: the true effect is likely to be substantially different from the estimated effect.
